# Myosin individualized: single nucleotide polymorphisms in energy transduction

**DOI:** 10.1186/1471-2164-11-172

**Published:** 2010-03-15

**Authors:** Thomas P Burghardt, Kevin L Neff, Eric D Wieben, Katalin Ajtai

**Affiliations:** 1 Department of Biochemistry and Molecular Biology, Mayo Clinic Rochester, 200 First Street SW, Rochester, MN 55905, USA; 2 Department of Physiology and Biomedical Engineering, Mayo Clinic Rochester, 200 First Street SW, Rochester, MN 55905, USA

## Abstract

**Background:**

Myosin performs ATP free energy transduction into mechanical work in the motor domain of the myosin heavy chain (MHC). Energy transduction is the definitive systemic feature of the myosin motor performed by coordinating in a time ordered sequence: ATP hydrolysis at the active site, actin affinity modulation at the actin binding site, and the lever-arm rotation of the power stroke. These functions are carried out by several conserved sub-domains within the motor domain. Single nucleotide polymorphisms (SNPs) affect the MHC sequence of many isoforms expressed in striated muscle, smooth muscle, and non-muscle tissue. The purpose of this work is to provide a rationale for using SNPs as a functional genomics tool to investigate structurefunction relationships in myosin. In particular, to discover SNP distribution over the conserved sub-domains and surmise what it implies about sub-domain stability and criticality in the energy transduction mechanism.

**Results:**

An automated routine identifying human nonsynonymous SNP amino acid missense substitutions for any MHC gene mined the NCBI SNP data base. The routine tested 22 MHC genes coding muscle and non-muscle isoforms and identified 89 missense mutation positions in the motor domain with 10 already implicated in heart disease and another 8 lacking sequence homology with a skeletal MHC isoform for which a crystallographic model is available. The remaining 71 SNP substitutions were found to be distributed over MHC with 22 falling outside identified functional sub-domains and 49 in or very near to myosin sub-domains assigned specific crucial functions in energy transduction. The latter includes the active site, the actin binding site, the rigid lever-arm, and regions facilitating their communication. Most MHC isoforms contained SNPs somewhere in the motor domain.

**Conclusions:**

Several functional-crucial sub-domains are infiltrated by a large number of SNP substitution sites suggesting these domains are engineered by evolution to be too-robust to be disturbed by otherwise intrusive sequence changes. Two functional sub-domains are SNP-free or relatively SNP-deficient but contain many disease implicated mutants. These sub-domains are apparently highly sensitive to any missense substitution suggesting they have failed to evolve a robust sequence paradigm for performing their function.

## Background

Single nucleotide polymorphisms (SNPs) are common single base DNA sequence variants that account for a sizable portion of the genetic variability between individuals. Some SNPs are common and have minor allele frequencies that approach 50%, while others are found much less frequently. There is some conceptual overlap between rare SNPs (with minor allele frequencies of less than 1%) and disease implicated mutations, but in common usage the term polymorphism is restricted to non-pathogenic sequence changes. Genome SNP patterns are fingerprints identifying subpopulations with common heritage that potentially instigate subpopulation specific traits exploitable for individualized treatment of disease. For the purposes of this discussion, it is implicit that the SNPs accumulate in the genome because they negligibly affect survival, however, we recognize that some SNPs may in fact have deleterious effects on the expression or function of protein products.

Another distinction that is germane to the present work is between synonymous and nonsynonymous SNPs. SNPs that lie within the coding region are synonymous if they do not change the amino acid specified by the codon and are nonsynonymous when they do. Even though synonymous SNPs will not change protein sequence they might still impact expression of a protein product by influencing the subcellular localization, stability or translational efficiency of mRNA [1,2]. The nonsynonymous SNP introduces a single amino acid change into the protein sequence. Assessing the impact of such protein sequence changes on protein function can be difficult. For most SNPs whose minor allele frequency is at least 5%, it is likely that their impact on genetic fitness is minor [3]. SNPs that clearly have harmful functional consequences only rarely become more common than this 5% level in the population as a whole. For less common SNPs, homozygous individuals that have two copies of the SNP are rare and deleterious consequences can often be masked by the presence of the more common functional allele. Another factor in deciding the significance of the nonsynonymous SNPs is genetic redundancy. The affected gene could be one copy among several present in the genome. These factors decide the prevalence of the affected protein in the tissues and may be more important for survival than how the SNP affects protein function. Furthermore, a SNP induced loss of functionality can sometimes be compensated by redundant functionality in the larger physiological system. An example is skeletal muscle where the relative composition of the myosin isoforms depends on environmental factors such as exercise [4-6] or zero gravity [7]. The muscle tissue may adapt to loss of function in the SNP affected isoform to assure survival. Finally, while SNPs accumulated in the data base are identified world wide without apparent prejudice, they are not necessarily a random sampling of the genome. SNP frequency in different gene regions could reflect uneven DNA sequencing accuracy and efficiency or other unknown biases inadvertently built into the data base. Our results are implications from the current data base that changes and grows daily.

Here we identify and discuss the myosin heavy chain nonsynonymous SNPs. We will include even scarce SNPs where homozygous individuals are rare. We do so in the spirit of being comprehensive, with the full understanding that neutrality of function cannot be assumed in these cases. Additionally, some SNPs discussed appear in the database, but have not been confirmed by other groups. Our purpose is to provide a rationale for using these reports to investigate structure-function relationships in myosin. Functional sub-domains within myosin have emerged as disturbance propagators essential to transduction. They are structural entities in which to identify SNP substitution sites and focus speculation on how SNPs influence myosin function.

The myosin heavy chain (MHC) consists of a globular head domain called subfragment 1 (S1) and C-terminal tail responsible for dimerization and cargo binding. In muscle, myosin dimers form (thick) filaments with interacting tails and head domains projecting outward from the filament to associate with complementary actin (thin) filaments in a muscle sarcomere [8]. Cellular myosins have several forms [9-12]. The cellular myosin V dimer is a processive motor utilizing the two heads cooperatively to move along actin filaments [13].

**Figure 1:**
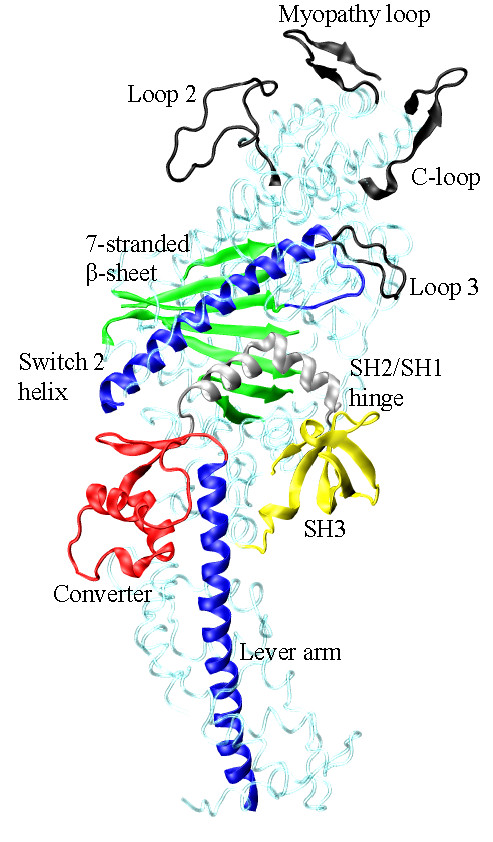
**Several myosin peptides or domains identified with energy transduction of ATP hydrolysis free energy to the mechanical work of moving actin**. Structured (Myopathy and Cloop, AA363-377 and 404-417) and unstructured (Loops 2 and 3, AA626-651 and 568-580) surface loops are actin binding peptides (black). The 7-stranded β-sheet (green, AA116-127, 170-180, 248-257, 265-271, 458-468, and 671-678) mediates ATP hydrolysis and actin binding affinity modulation. The Switch 2 helix (blue, AA469-509) transmits translational movement in the active site to the converter domain (red, AA716-772) where it is converted to the torque needed to rotate the lever-arm (blue, AA773-813). The SH2/SH1 hinge (silver, AA688-715) undergoes a large conformation change with lever-arm rotation. The function of SH3 (yellow, AA30-80) in energy transduction is not yet understood. Not depicted are actin-binding-peptides at two sites in the upper half of the molecule.

S1 contains functional sub-domains that retain their structure, but move relatively, during transduction [14]. ATPase transduction starts in the active site composed in part by the P-loop, Switch 1, and Switch 2 polypeptides (linked to a 7-stranded β-sheet separating the active site from the actin binding site) that sense the γ-phosphate position and coordination. ATP hydrolysis in the active site is not followed immediately by phosphate release because the Switch 1 R246 and Switch 2 E469 salt-bridge "back door" inhibits phosphate release until actin binds. With actin binding, the back door opens to release phosphate and the Switch 2 α-helix (or relay helix) transmits linear force originating from the active site to a converter domain converting linear force into torque to rotate the lever-arm. The lever-arm α-helix, rigidified by the bound myosin light chains (MLCs), amplifies displacement and impels myosin relative to actin [15,16]. A region between the switch 2 helix and the converter domain, the SH2/SH1 hinge, undergoes substantial conformation change during the transduction. The 7-stranded β-sheet, switch 2 helix, converter, lever-arm, and SH2/SH1 hinge are the sub-domains identified in the skeletal myosin crystal structure depicted in Figure 1[17].

Multiple peptides within S1 (C-loop, Myopathy loop, and others) constitute the actin binding site. This site alternates between weak and strong actin binding affinity in coordination with the lever-arm swing. Phosphate release in muscle myosin ATPase (or the opening of the R246/E469 back door salt-bridge) is rate limiting due to its inhibition by a large entropic energy barrier in the absence of actin. Phosphate release initiates lever-arm swing hence the rate limiting step inhibits progress through the cycle until actin binds and a work producing lever-arm power stroke is possible. Coupling actin binding to reducing the energy barrier to phosphate release is an allosteric effect predicted for Myopathy or C-loop structured surface loops [18,19]. We showed the C-loop is the allosteric actin contact sensor mediating bidirectionally between the actin-binding and active sites [20]. The C-loop lowers the energy barrier to phosphate release by coupling structural transitions within the S1 that allow phosphate release without inducing strain in the 7-stranded β-sheet. Several actin binding myosin surface loops are identified in Figure 1.

In this paper we locate nonsynonymous SNPs within the functional sub-domains and evaluate their expected functional impact based on: i) how well the native residue is conserved among isoforms, ii) comparison of general physical characteristics (size, charge, hydrophobicity, etc.) between the native and substituted side chain, and iii) (when possible) the hypothetical role of the native residue in transduction and how this role could be affected by the substituted side chain. We find that SNPs infiltrate *nearly *every corner of the myosin head and that their significance may lie in where they are tolerated in contrast to where they are excluded.

## Results

Twenty-two human myosin heavy chain genes MYH1-4, MYH6-15, MYH7B, MYOIE, MYO6, MYO7A and 7B, MYO9A and 9B, and MYO10 were searched for nonsynonymous SNPs as described in METHODS. We confined the search to SNPs in the S1 domain. Table 1 summarizes results with the SNP reference number locating it in the human genome and in the NCBI data base, the sequence position relative to the skeletal myosin 2× sequence, the MHC gene and myosin isoform it encodes, the position for the SNP within myosin domains, the average percentage of heterozygous genes (one allele normal the other SNP substituted) in the standard sub-populations, whether (Yes or No) there are SNP substituted homozygous genes (both alleles SNP substituted) in the standard sub-populations or observed in screened individuals, and the number of individuals screened. Missing data is denoted with a hyphen.

**Table 1:** Myosin heavy chain SNPs^a^

SNP (rs number)	Skeletal 2× sequence	Skeletal 2× residue if different	Gene	Isoform	Position^b^	**<Hetero>^c ^(Homo-zygotes?) **^e^	Count^d^
**7-stranded β-sheet (Figure 2)**							
61745307	I88M		MYH13	super-fast	near β_1_	-	-
442275	E89Q		MYH6	α-cardiac	near β_1_	0.5(N)	525
61739660	H98Q		MYH1	2×	near β_1_	-	-
2754166	D108E	E108	MYH7	β-cardiac	near β_1_	0(N)	525
7737765	H170Y	R170	MYO10	myosin X	β _4_	-	-
61745058	D171E	E171	MYH7B	β-cardiac	β _4_	-	-
61745059	N172D		MYH7B	β-cardiac	β _4_	-	-
45516091	R240W		MYH7	β-cardiac	SW1	0(Y)	0^f^
28934610	R246H		MYO7A	myosin VIIA	SW1	-	-
3218713	R252Q (disease implicated)		MYH7	β-cardiac	β _6_	0(Y)	525
55645295	I253V		MYH14	iso1/iso2	β _6_	-	-
28936390	E257V	T257	MYO6	myosin VI	β_6_	-	-
34416201	T258K		MYH13	super-fast	β _6_/β _7_	2.5(N)	39
4299484	R445Q		MYH15	unknown	near β_7_	18.0(?)	1178
61731179	N447K		MYH6	α-cardiac	near β_7_	-	-
61745057	L463P		MYH7B	β-cardiac	β _5_	-	-
4981473	E469Q		MYH7	β-cardiac	SW2	0(N)	525
28932773	R675Q (disease implicated)		MYH8	perinatal	β _3_	-	-
11539755	E681K		MYOIE	myosin 1e	near β _3_	-	-
**Switch 2 helix (Figure 4)**							
61734198	Q478H		MYH11	sm1A/sm2B	SW2 hx	-	-
1064307	Y483H	F483	MYO9B	myosin IXB	SW2 hx	-	-
28934903	N485I		MYO7A	myosin VIIA	SW2 hx	-	-
61745301	H494D		MYH13	super-fast	SW2 hx	-	-
9868484	H495Y		MYH15	unknown	SW2 hx	38.3(Y)	1121
1052031	F497L		MYO7A	myosin VIIA	SW2 hx	-	-
3218715	E502K (disease implicated)		MYH7	β-cardiac	SW2 hx	0(Y)	525
3729813	K505N		MYH7	β-cardiac	SW2 hx	0(N)	525
**SH2/SH1 hinge, converter, & lever-arm (Figure 3)**							
73974725	M688V		MYH1	2×	SH2/SH1 hn	-	-
34515627	V694E	L694	MYO7A	myosin VIIA	SH2/SH1 hn	-	-
41298143	R695H	H695	MYO7A	myosin VIIA	SH2/SH1 hn	-	-
2190729	G701R		MYH13	super-fast	swivel	2.8(Y)	384
28940307	R707S		MYH14	iso1/iso2	SH2/SH1 hn	-	-
3181426	R710S		MYH7	β-cardiac	SH2/SH1 hn	-	-
35641839	V720I (disease implicated)		MYO7A	myosin VIIA	Converter	-	-
1136661	Y723C(disease imp)K723		MYO10	myosin X	Converter	-	-
3746442	P735S (disease implicated)		MYH7B	β-cardiac	Converter	^g^	525
26740	R743Q	K743	MYO10	myosin X	Converter	13.5(?)	1122
11847151	L785M		MYH6	α-cardiac	Lever-Arm	0(N)	525
36090425	D787N	Q787	MYO7A	myosin VIIA	Lever-Arm	-	-
3218716	A801T (disease implicated)		MYH7	β-cardiac	LA & ELC IQ	0(Y)	690
**SH3 (Figure 5)**							
17092199	P31T		MYH7B	β-cardiac	SH3	discordant(?)^h^	1013
28711516	G57R		MYH6	α-cardiac	SH3	-(Y)	-
17707947	V60I		MYO10	myosin X	SH3	12.9(?)	1092
41309316	K68N	A68	MYH7B	β-cardiac	SH3	-	-
**Actin binding (Figure 6)**							
45629132	R370H	K370	MYO7A	myosin VIIA	C-loop	-	-
35222064	Q371P		MYH13	super-fast	C-loop	2.5(N)	39
2515926	P378Q		MYH6	α-cardiac	C-loop	-	-
3218714	R406W (disease implicated)		MYH7	β-cardiac	Myopathy	0(Y)	525
45522831	R408C	K408	MYH7B	β-cardiac	Myopathy	-	-
35349985	F534L		MYH4	2b	actin bind	2.8(N)	39
59922029	R570K	K570	MYO10	myosin X	Loop 3	-	-
61735348	V572A/D	A572	MYH3	embryonic	Loop 3	-	-
28565077	A621V		MYH15	unknown	near Loop 2	-	-
61745053	T631N	E631	MYH7B	β-cardiac	Loop 2	-	-
34693726	A637V	G637	MYH8	perinatal	Loop 2	9.2(N)	310
2276282	E643K	K643	MYO7A	myosin VIIA	Loop 2	-	-
61743282	F655S		MYO7B	myosin VIIB	actin bind	-	-
**N-term, U50, and L50**							
3729993	D4A		MYH7	β-cardiac	N-term	0(Y)	525
34042358	D4E		MYH13	super-fast	N-term	3.2(?)	62
45511396	R18C		MYH7	β-cardiac	N-term	0.4(Y)	0^f^
61730792	R24P/L		MYH1	2×	N-term	-	-
41312286	T25M	I25	MYH7B	β-cardiac	N-term	-	-
590722	P(<32)T^i^	-	MYH14	iso1/iso2	undefined	-	-
2404991	G(<48)S^i^	-	MYO7B	myosin VIIB	undefined	-	-
1052030	L(<58)S^i^	-	MYO7A	myosin VIIA	undefined	-	-
35218876	R(<64)Q	-	MYO9B	myosin IXB	undefined	2.5(?)	39
10518970	P(<82)L^i^	-	MYO9A	myosin IXA	undefined	4.3(?)	1224
17855105	R(<82)K^i^	-	MYO9A	myosin IXA	undefined	-	-
2929516	T(<82)I^i^	-	MYO9A	myosin IXA	undefined	discordant(?)^h^	1122
34773557	M142I	A142	MYH14	iso1/iso2	N-term	2.8(?)	70
6174305	R145G	G145	MYO10	myosin X	N-term	-	-
41298131	I148T	R148	MYO7A	myosin VIIA	N-term	-	-
58359270	L199F	I199	MYO9B	myosin IXB	N-term	-	-
28936391	H287R		MYO6	myosin VI	U50	-	-
6870170	E300D	L300	MYO10	myosin X	U50	1.3(?)	908
35512085	T307P		MYH4	2b	U50	-	-
34498817	P320A	E320	MYH14	iso1/iso2	U50	5.5(?)	309
35315400	S323C	V323	MYH14	iso1/iso2	U50	2.5(?)	78
34124921	I326T		MYH8	perinatal	U50	1.2(N)	39
35984286	Q329R (disease implicated)		MYH4	2b	U50	2.5(N)	39
34846075	V335I	T335	MYO10	myosin X	U50	2.5(?)	39
41298135	R336H	D336	MYO7A	myosin VIIA	U50	-	-
34419805	T345A		MYH8	perinatal	U50	2.8(N)	35
61756677	V350D/A		MYH2	2a	U50	-	-
11750538	R350W	V350	MYO10	myosin X	U50	50(?)	1264
1724577	E389D	Y389	MYH12	myosin V	U50	17.2(Y)	1262
61732664	Y(389-390)H^j^		MYO6	myosin VI	U50	-	-
61742021	I514T (disease implicated)		MYO7B	myosin VIIB	L50	-	-
12949680	A594T		MYH4	2b	L50	7.4(N)	39

^a ^Abbreviations SW1 or SW2 denote Switch 1 or 2, hx is helix, sb is salt-bridge, hn is hinge, and hyphen (-) is

no data submitted.

^b ^Position names are defined in the MHC sequence as follows:

1. SH3 like β-barrel: AA30-80

2. 7-stranded β-sheet: β_1 _AA116-120; β_2 _121-127; β_3 _671-678; β_4 _170-180; β_5 _458-468; β_6 _248-257; β_7 _265-271

3. Loop 1 AA200-220

4. Upper 50 k (U50) AA220-468

5. C-loop AA363-377

6. Myopathy loop AA404-417

7. Switch 2 helix AA469-509

8. Lower 50 k (L50) AA469-620

9. Actin binding peptides: hydrophobic helical peptides AA529-560 and 652-661, Loop 2, Loop 3,

C--loop, Myopathy loop

10. Loop 3 actin binding site: AA568-580

11. Strut loop: AA600-604

12. Loop 2 AA626-651

13. SH2/SH1 hinge AA688-715

14. Converter domain: AA716-772

15. Lever-arm AA773-813

^c^Average percentage of heterozygous genes (one allele normal, the other SNP substituted) in the standard subpopulations.

^d ^Individuals sequenced.

^e ^Are there SNP substituted homozygous genes (both alleles SNP substituted) in the standard sub-populations or

observed in screened individuals?

^f ^Unexplained inconsistency.

^g ^All individuals sequenced are homozygous in the SNP replacement over 4 population groups.

^h ^Discordant genotype implies conflicting reports.

^i ^Sequence segment not aligned in BLAST.

^j ^Sequence segment SLEYCAE in myosin VI inserts between AA389 and 390 in MYH1. This is not Insert 1 or

2 mentioned in the text.

Most MHC isoforms contribute SNPs including the fast skeletal muscle isoforms 2×, 2a, 2b, embryonic, and perinatal; α- and β-cardiac myosins; smooth muscle variants sm1A and sm2B; myosin V; super-fast myosin; two isoforms of MYH14; MYH15; and other unconventional myosins including IE, VI, VIIA, VIIB, IXA, IXB, and X. Myosin 2b (MYH4) is a fast skeletal myosin isoform apparently expressed in a skeletal jaw closing (masseter) muscle [21]. The embryonic and perinatal isoforms (MYH3 and 8) are developmentally regulated [22,23]. The embryonic isoform also expresses in regenerating muscle and in extraocular muscles (EOM) [24]. A mutation in perinatal myosin is implicated in a rare genetic disease causing distal arthrogryposis syndrome [25]. The α-cardiac isoform (MYH6) is predominantly expressed in the atrium [26]. The β-cardiac isoform (MYH7) is predominantly expressed in the heart ventriculum and in slow or type 1 skeletal muscle fibers. Numerous heart disease implicated mutations occur in the β-cardiac MHC [27]. Another myosin isoform (MYH7B/MYH14) on chromosome 20 is called a β-cardiac myosin with 85% homology with the β-cardiac myosin coded by MYH7.

The MYH11 gene encodes the 4 smooth muscle myosin isoforms that differ near the C-terminus (isoforms 1 and 2) or at Loop 1 where a seven amino acid peptide is omitted (isoform A) or inserted (isoform B) [28]. The Loop 1 insert correlates with tissue localization [29] and enzyme kinetics [30,31]. Nucleotide-induced fluorescence intensity increase in the highly conserved W511 occurs in all but one myosin isoform implying it results from common origin within the conserved motor core. The smA isoform has nucleotide-induced tryptophan fluorescence enhancement like other myosin isoforms but we showed with site-directed mutagenesis that the contributing tryptophan is probably not W511. These results suggested that smA and smB myosin isoforms have different influence propagating pathways emanating from the active site through the switch 2 helix containing W511 to the converter domain [32].

Processive myosin V (MYH12) translates over actin filaments using a hand-over-hand mechanism [33-35]. The super-fast isoform (MYH13) expresses in EOMs performing diverse functions including eye movement [24]. The EOMs are more complex than limb muscles with 9 or more different MHCs expressed in adult muscle including the fast skeletal, β-cardiac, and embryonic MHC. The super-fast myosin supports higher velocity and lower tension contractions [36]. The MYH14 gene codes for 2 myosin isoforms (iso1/iso2 in Table 1) expressed in muscle and non muscle tissue. The MYH14 gene is implicated in hearing disorders [37]. SNP variants in MYH15 are associated with elevated risk for heart disease [38].

**Scheme 1:**
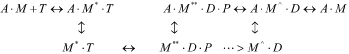


Myosin IE (MYOIE) is a cellular myosin with an extended C-loop thought to interact with tropomyosin in tropomyosin-containing actin [39,40]. Myosin VI is a reversedirection motor with core structure resembling forward-direction motors except for two inserts (1 & 2). Insert 1, near the active site and Switch 1, is probably partially responsible for the slow ADP release (A·M^·D→A·M in Scheme 1, METHODS)[41]. Insert 2 modifies the converter domain to re-direct lever-arm swing for reverse-directed motility [41,42]. Mutations in myosin VII Aassociate with Us her syndrome that in its most severe phenotype is a deafness-blindness disorder [43,44]. Myosin IXB is a single headed processive motor that is unique because its rate limiting step is not ADP release but ATP hydrolysis (M*·T→M**·D·P in Scheme 1) suggesting it remains actin bound even in a weak actin binding state [45]. Slow dissociation from actin is attributed to tethering by a unique Loop 2 insertion. Myosin X is a membrane associated motor involved in filopodia motility [46] that has novel monomeric and dimeric conformations at physiological protein concentrations [11]. Calmodulin or calmodulin like protein (CLP) are the lever-arm bound myosin light chains in myosin X [47]. Myosin X is believed to be processive consistent with its function in the cell.

### SNP implications for motor function

**Figure 2:**
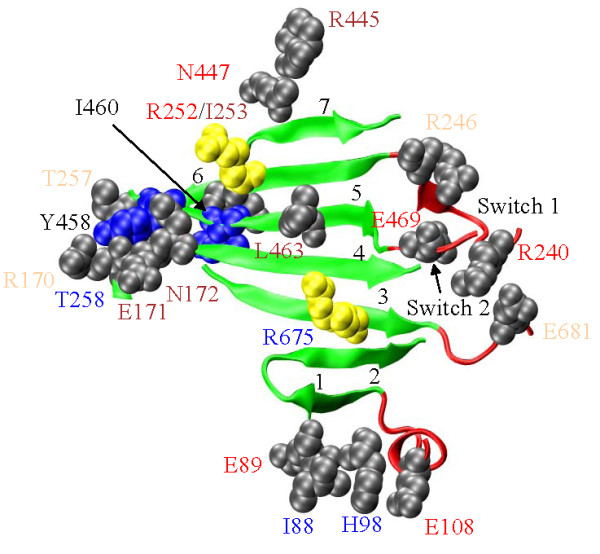
**The 7-stranded β-sheet (green) and the adjacent structures (red)**. SNP substitution sites in the vicinity are depicted in grey or yellow with space filling models of the unsubstituted side chains. The yellow residues are also implicated in disease. SNP residue annotation color coding corresponds to: cardiac myosin II (**red**), skeletal myosin II (**blue**), non-muscle myosin II (**brown**), and unconventional myosin (**tan**). Residues annotated in black are not SNPs. The 7 strands are numbered at the tip indicating the sequence number increasing direction. R675 on β_3 _(AA671-678); R170, E171, and N172 on β_4 _(AA170-180); L463 on β_5 _(AA458-468); and R252, I253, and T257 on β_6 _(AA248-257) modify the structure. E681 is three residues past the end of β_3_, E469 is on Switch 2 just two residues past the end of β_5_, R240 and R246 are on Switch 1 just before the start of β_6_, and T258 lies between β_6 _and β_7 _(AA265-271). Residues I88, E89, H98, E108, R445, and N447 are distant in sequence but spatially associated with the 7-stranded β-sheet structure. E89 is the most distant at ~7.5 angstroms. Blue side chains for residues Y458 and I460 are not SNPs but are proposed to contribute substantially to the energy barrier determining the rate limiting step to myosin ATPase product release in the absence of actin.

**Figure 3:**
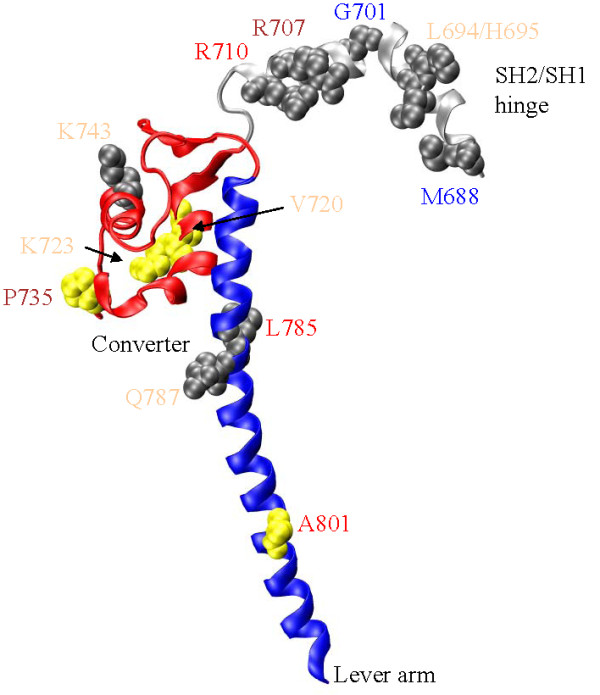
**The SH2/SH1 hinge (silver, AA688-715), converter (red, AA716-772), and lever-arm (blue, AA773-813)**. SNP residue annotation color coding corresponds to: cardiac myosin II (**red**), skeletal myosin II (**blue**), non-muscle myosin II (**brown**), and unconventional myosin (**tan**). Thirteen SNP substitution sites at M688, L694, H695, G701, R707, R710, V720, K723, P735, K743, L785, Q787, and A801 are depicted in gray or yellow with space filling models of the unsubstituted side chains. The yellow residues are also implicated in disease. R710 is adjacent to the reactive thiol residue (SH1 at C709) on the SH2/SH1 hinge. L785, Q787, and A801 are on the lever-arm and within the ELC binding region. The converter domain receives impulses from the Switch 2 helix that it converts into the torque needed rotate the lever-arm while the SH2/SH1 hinge changes conformation.

Three SNPs, R246H, E469Q (Figure 2) and G701R (Figure 3), are remarkable because of the functional significance of the residues they modify. R246 and E469 form the Switch 1/Switch 2 salt-bridge at the γ-phosphate in bound ATP preventing product release following hydrolysis in the active site of S1. Site-directed mutagenesis of *Dictyostelium discoideum *(Dicty) myosin produced R246E, E469R, and the double mutant R246E/E469R reversing the polarity of the salt-bridge [48]. Only the double mutant remained functional but with altered ATPase kinetics. Similar experiments using expressed smooth muscle myosin suggested R246 is significant for back door closure after ATP binding to the active site and that E469 is important for ATP hydrolysis by its positioning of a water molecule for nucleophilic attack on the γ-phosphate [49]. The E469Q SNP substitution does not necessarily prohibit the latter mechanism although E469A/R mutants eliminated the phosphate burst and actin-activated ATPase. The R246H SNP substitution alters size but not charge of the side chain while R246A/E mutants likewise eliminated the phosphate burst and actin-activated ATPase. The loss of the phosphate burst and actin-activated ATPase indicates the phosphate release step is no longer rate limiting undermining the efficient energy transduction in muscle because the ATPase cycle disengages from the work producing interaction with actin. The SNPs suggests it would be interesting to test the R246H and E469Q substitutions in an in vitro system. R246H occurs in myosin VIIA where ATPase is rate limited by ADP release rather than phosphate release possibly explaining its tolerance to substitution. E469Q occurs in β-cardiac myosin where ATPase is rate limited by phosphate release. R246H and E469Q occur with unknown or apparently low probability for expression, respectively.

G701R in super-fast myosin replaces the glycine swivel with a residue containing a bulky side chain inhibiting free rotation in the Ramachandran angles that is characteristic only to glycine [50]. The highly conserved G701 pivot was found to be essential for motor activity in skeletal [51] and Dicty [52] myosins. S1 crystal structures depicting myosin conformation in M** [53] and M* [17] lever-arm orientation states indicate G701 swivels 40-50 deg about two axes with the lever-arm swing [54]. Unlike nearby swivel-candidate residues, G705 and G712, the G701 maintains conformations in the pre- and post-powerstroke states that only glycine can readily accommodate. If the G701R substituted protein truly functions in healthy individuals, it suggests the super-fast myosin has a substantially modified transduction mechanism to accomplish its high velocity contraction. The G701R substitution occurs with an average heterozygous population in percent (AHP) of 2.8% and with homozygous individuals identified.

Figure 2 shows the 7-stranded β-sheet separating the active from the actin binding sites and numerous residue positions with SNP substitutions. Substitutions H170Y (R170 in MYH1), D171E (E171 in MYH1), and N172D modify β_4_; L463P modifies β_5_; while I253V and E257V (T257 in MYH1) modify β_6 _within the β-sheet structure. SNP substitution R675Q modifying β_3 _is implicated distal arthrogryposis syndrome [25] and in heart disease together with SNP substitution R252Q modifying β_6 _[27]. The R/Q substitution replaces a large +charged side chain with a polar, neutral, and smaller side chain where total surface area changes by ~52 Å^2^. I/V, D/E, H/Y and N/D substitutions are conservative of charge, size, and polarity. E/V substitution is not conservative of charge, size, or polarity. The L/P substitution could be the most perturbative due to proline's backbone restrictive geometry inducing strain in the β-sheet. Distortions in this β-sheet accompany events associated with ATP hydrolysis and product release since the P-loop, Switch 1, and Switch 2 are closely linked with the β_4_, β_6_, and β_5 _strands, respectively [55]. Furthermore, crystal structures of myosin V without bound nucleotide models the actin-attached rigor conformation and suggests strong binding with actin closes a cleft within the 50 kDa domain of S1 [56] causing distortions in the β_1_, β_2_, and β_3 _strands [55]. Thus many aspects of energy transduction impact the 7-stranded β-sheet and in particular, strands 3-6 containing the SNP substitutions. Our MCMD simulation suggests distortion of the β-sheet in the β_5 _and β_7 _strands provides a substantial part of the entropy dominated energy barrier to product release in the absence of actin [19]. In particular, β_5 _residues Y458 and I460 (Figure 2) have solvent accessible surface areas (ASA) that undergo dramatic increases with barrier transition contributing significantly to the entropy dominated free energy barrier. Simulation suggests residues on β_5 _and β_7 _should more likely perturb the contraction mechanism implying the SNP substitution L463P modifying β_5 _may again have special significance. These SNPs occur in a variety of myosin genes and isoforms but most (including L463P) are relatively new entries that have not undergone further verification to test their presence in sub-population data bases.

Figure 2 also shows Switch 1 and the R240W and R246H SNP sites adjacent to the 7-stranded β-sheet, E681K adjacent to β_3_, Switch 2 and the E469Q SNP site adjacent to β_5_, T258K appearing between the β_6 _(AA248-257) and β_7 _(AA265-271) strands, and D108E (E108 in MYH1) upstream from β_1 _and adjacent to the 7-stranded β-sheet. Additionally, I88M, E89Q, and H98Q are within a few angstroms of β_1_, while R445Q and N447K are within a few angstroms of β_7_. The R240W substitution provides an opportunity to insert a harmless spectroscopic probe (i.e., W240) into the Switch 1 peptide to sense directly the back-door dynamics and induced strain in the 7-stranded β-sheet. The D108E SNP reiterates the natural cardiac/skeletal myosin isoform sequence substitution. I88M, E89Q, and H98Q connect the 7-stranded β-sheet to SH3. N447K appears within a cluster of heart disease linked mutations [27]. R445Q has an AHP of 18% and comes from MYH15. The next largest AHP SNP at 2.5% from around the 7-stranded β-sheet is T258K in super-fast myosin. Seventeen SNP substitutions (excluding disease linked R252Q and R675Q) in, or proximal to, the 58 residue 7-stranded β-sheet is unexpected given the significance attributed to the region.

**Figure 4:**
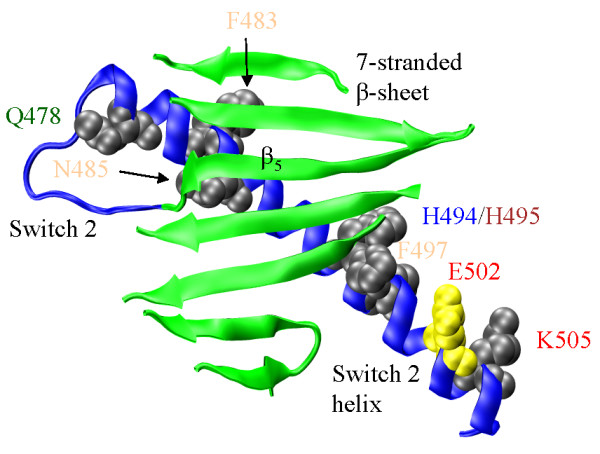
**The 7-stranded β-sheet (green), Switch 2, and the Switch 2 helix (blue)**. SNP residue annotation color coding corresponds to: cardiac myosin II (**red**), skeletal myosin II (**blue**), smooth muscle myosin II (**green**), non-muscle myosin II (**brown**), and unconventional myosin (**tan**). Eight SNP substitution sites at Q478, F483, N485, H494, H495, F497, E502, and K505 on the Switch 2 helix are depicted in gray or yellow with space filling models of the unsubstituted side chains. The yellow residue is also implicated in disease. Movement at Switch 2 propagates to the Switch 2 helix and to the converter domain (not shown) via the tip of the helix near K505.

Figure 4 shows the Switch 2 helix underlying the 7-stranded β-sheet where β_5 _and the Switch 2 helix are linked by Switch 2, and sites for the eight SNP substitutions, Q478H, Y483H (F483 in MYH1), N485I, H494D, H495Y, F497L, and K505N, modifying the Switch 2 helix. SNP substitution E502K is implicated in heart disease [27]. The Switch 2 α-helix transmits linear force originating from the active site to the converter domain. Helix rigidity could be essential to force transmission. Q478 is a highly conserved residue at the beginning of the helix where it could maximally affect force transmission efficiency by reducing rigidity. The Q478H substitution tests this hypothesis because the Q/H substitution is helix destabilizing [57] suggesting helix stability may not be crucial. Other SNP substitutions, N485I, H495Y, F497L and E502K, are helix stabilizing while Y483H, H494D and K505N are destabilizing. K505N is at the C-terminal end of the helix where destabilization would be least detrimental. H495 is a potentially sensitive point where the helix develops a kink in the pre-power stroke M** conformation [53]. The H495Y substitution from MYH15 has an AHP of 38% and homozygous individuals were identified. A H495W mutation might sense Switch 2 helix movement by tryptophan fluorescence changes and represents a desirable signal donor in the transduction mechanism. Highly conserved residues on the tip of the switch 2 helix, Y504 and I509, are thought to mediate interaction with the converter domain but are not affected by known SNPs.

Figure 3 shows the SNP substitutions sites for the SH2/SH1 hinge [M688V, V694E (L694 in MYH1), R695H (H695 in MYH1), G701R, R707S, R710S], converter [R743Q (K743 in MYH1)], and lever-arm [L785M and D787N (Q787 in MYH1)]. SNP substitutions V720I, Y723C, and P735S in the converter, and A801T in the lever-arm, are implicated in heart disease [27]. The converter domain position P735 is implicated is heart disease by a P735L substitution in β-cardiac MHC [27]. The P735S SNP replacement is homozygous in the 4 subpopulation groups tested suggesting the reference sequence is incorrect.

The converter domain has been implicated as the element undergoing elastic distortion in the myosin accompanying force development prior to mechanical work production [58,59]. Site directed mutagenesis in the SH2/SH1 hinge--converter interface, and in the converter domain proper, provided clues for how it performs the conversion of linear translation in the Switch 2 helix to the rotation of the lever-arm [60]. Mutations F713A or F768A completely eliminated or sharply inhibited the ability of smooth muscle myosin to function mechanically. Standard kinetics experiments looking at the ATP binding, hydrolysis, product release, and the fluorescence from the ATP sensitive tryptophan (W511) indicate active site kinetics and transmission of force through the Switch 2 helix are fully intact in the mutants. The findings suggest that the mutations perturb only the converter function. Other experiments, specifically fluorescence resonance energy transfer (FRET), actin sliding assay, and single myosin force development show that both mutants are compromised mechanically. The F713A mutant causes a catastrophic mechanical failure due to the loss of the hydrophobic contact between the SH2/SH1 hinge and converter. The F768A mutant looses the rigidity of the Switch 2 helix/converter contact at F768 making a motor that can rotate its lever-arm but with less force. The SH2/SH1 hinge has a high density of SNP substitutions with 6 occurring in a 28 residue peptide. SNP sites at R707 and R710 are highly conserved among myosin isoforms but do not appear to enter into the crucial interaction with the converter domain. M688 is not conserved among the myosin isoforms.

The sole converter domain SNP substitution not implicated in disease is R743Q that is also common with an AHP of 13.5%. The SNP occurs in myosin X that has a low converter domain sequence homology with myosin 2×. Myosin VI, VII, and X have a unique structure at the head-tail junction wherein a monomeric single α-helix (SAH) competes with the dimeric coiled-coil [9-11]. The SAH domain might function as an elastic element undermining converter domain significance in this isoform. Hypothetically, the converter domain is sensitive to mutation because it usually needs to perform the two functions of the linear-to-rotary motion converter and elastic element.

The L785M, D787N (Q787 in MYH1), and A801T SNP substitutions modify the leverarm. Mutation in this region could interfere with essential light chain (ELC) binding. The loss of light chains, either regulatory (RLC) or ELC, affects myosin morphology and its motor function probably by lowering the rigidity of the lever-arm domain [61]. Either substitution could also directly perturb the lever-arm structure and rigidity also leading to loss of function. None of these possibilities may be happening due to the lever-arm SNP substitutions because their consequences would be severe. Aside from G701R and R743Q, SNPs shown in Figure 3 affect a small or unknown percentage of the population.

**Figure 5:**
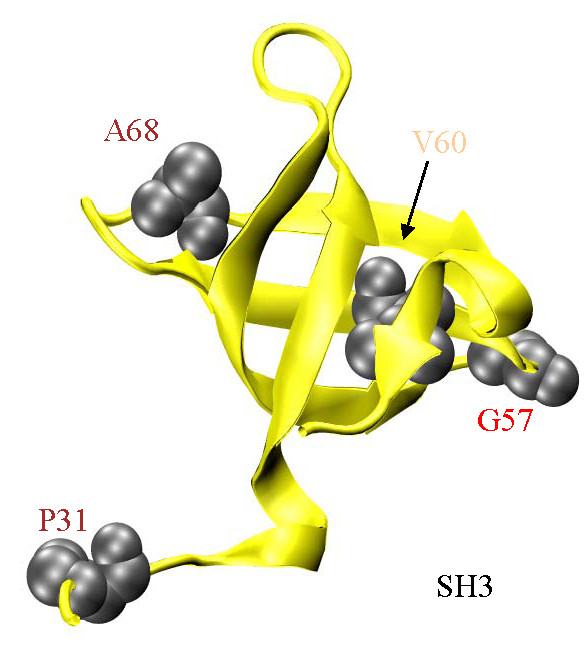
**The SH3 domain (yellow, AA30-80) and 4 SNP substitution sites at P31, G57, V60, and A68 are depicted in gray with space filling models of the unsubstituted side chains**. SNP residue annotation color coding corresponds to: cardiac myosin II (**red**), non-muscle myosin II (**brown**), and unconventional myosin (**tan**). The function of SH3 is unknown but it is implicated in transmission of influence from ELC to the active site and in supporting the binding of the ELC N-terminus to actin. Several SNP substituted residues adjacent to the 7-stranded β-sheet (**Figure 2**) are also adjacent to SH3 including I88, E89, H98, and E108.

Figure 5 shows the SH3 domain (AA30-80) and the sites for the P31T, G57R, V60I, and K68N (A68 in MYH1) substitutions. The SH3 domain is a β-barrel located close to the myosin active site but with uncertain function. The SH3 domain undergoes significant conformational change during ATP hydrolysis including at the G57 site where Ramachandran angle changes consistent with a glycine residue will be inhibited by steric clash with the arginine side chain. Smooth muscle myosin contains tryptophan residues in SH3 and its vicinity that sense conformational change in the N-terminal domain with nucleotide binding to the active site [62]. N-terminus truncated constructs of the Dicty S1 showed SH3 played a role is structural stabilization of S1 and in communication among functional domain within the myosin head [63].

The essential light chain (ELC) appears to couple SH3 and lever-arm sub-domains in S1. LC1 and LC3 are ELC isoforms expressed in skeletal muscle with the S1 heavy chain binding LC1/RLC or LC3/RLC pairs in equal amounts. LC1 differs from LC3 by a 40 residue extension at the N-terminus. Cardiac myosin has only LC1. The flexible LC1 N-terminus is not resolved in S1 crystal structures and is not shown in Figure 1. Electron cryomicroscopy reconstructions suggest SH3 and the N-terminus of LC1 interact in skeletal muscle myosin [64]. There it is suggested that the SH3/LC1 interaction could facilitate the (N-term)LC1/(N-term)actin interaction known to affect the overall actomyosin interaction in skeletal and cardiac myosins [65,66] and that SH3 could contain an LC1/active-site path of influence known to modulate myosin ATPase [67]. The P31T substitution in MYH14 and V60I in myosin X are common.

### SNP implications for actomyosin

**Figure 6:**
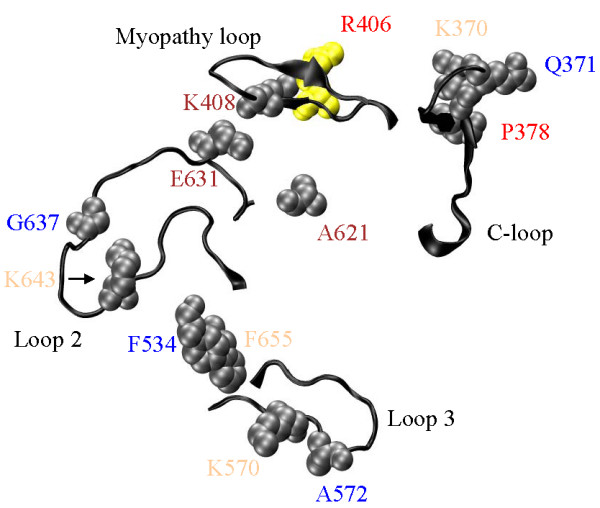
**Elements of the actin binding site on S1**. Structured surface loops (Myopathy and Cloop, AA404-417 and 363-377), unstructured surface loops (Loops 2 and 3, AA626-651 and 568-580), and F534 and F655 in the primary hydrophobic actin contacts (AA529-560 and 652-661) are actin binding peptides. SNP residue annotation color coding corresponds to: cardiac myosin II (**red**), skeletal myosin II (**blue**), non-muscle myosin II (**brown**), and unconventional myosin (**tan**). Thirteen SNP substitution sites K370, Q371 and P378 (C-loop); R406 and K408 (Myopathy loop); F534 and F655 (primary hydrophobic contact); K570 and A572 (Loop 3), and, A621, E631, G637, and K643 (Loop 2) are depicted in gray or yellow with space filling models of the unsubstituted side chains. The yellow residue is also implicated in disease.

Protein-protein contacts in actomyosin were identified by experimental structural studies combined with simulated docking of the myosin and actin crystal structures [68-72], by detecting changes in actin binding strength, actin-activated myosin ATPase, and in vitro motility caused by the mutation of small peptide segments or individual residues in myosin [73-81]. Primary hydrophobic actin contacts are helical segments, AA529-560 and AA652-661, while the unstructured surface Loop 2 (AA626-651) maintains ionic interactions with the actin N-terminus. Secondary sites are an unstructured surface loop, Loop 3 (AA568-580), and the structured Myopathy loop (AA404-417) [68] also on the S1 surface. Figure 6 shows the actin binding myosin surface loops including Loops 2, 3, Myopathy and C-loop. Also shown are actin binding site SNP substitutions F534L and F655S that fall within a primary hydrophobic actin contact.

Experimental work comparing tertiary structure of skeletal and β-cardiac MHC identified the C-loop (AA363-377), Figures 1 and 6, having influence on active site coupling to actin and possibly a direct interaction with actin [82]. Limited proteolysis of skeletal S1 cleaves the heavy chain at Loop 1 (AA200-220) and Loop 2 producing 25, 50, and 20 kDa molecular mass fragments [83]. These loops are involved in regulation of substrate release (Loop 1) and in actin binding and regulation of actin-activated ATPase (Loop 2) [75,78,84-86]. Limited proteolysis of βS1 cleaves the heavy chain at equivalent points and at the C-loop within the 50 kDa fragment [82]. The C-loop cleavage dramatically affects βS1 Mg^++^ATPase suggesting it participates in energy transduction [82]. Actin binding protected Loop 2 from proteolysis in skeletal S1 indicating Loop 2 involvement in actin binding [87,88]. Actin binding to βS1 fails to inhibit Loop 2 cleavage [82,89] but does inhibit C-loop cleavage [82]. These observations highlight differences in skeletal S1 and βS1 conformation and identify the C-loop as a possible actin binding site. The C-loop was proposed as a site of actin binding in skeletal S1 [71,72], in myosin V [90], and in myosin IE where it is called Loop 4 [39]. We constructed single site mutations in the smooth muscle myosin C-loop and chimeric proteins containing C-loop sequences from βS1 and skeletal S1 to investigate how sequence perturbs C-loop function [20,91]. Based on this work we proposed that the C-loop is an allosteric actin contact sensor initiating actin-activation of the myosin ATPase.

The C-loop SNP substitutions, R370H (K370 in MYH1), Q371P, and P378Q, occur in the myosin VIIA, super-fast myosin and α-cardiac myosin, respectively. In super-fast myosin, the Q/P substitution can be expected to perturb and rigidify the C-loop structure with the proline. We proposed structural rigidity in the C-loop is probably necessary for its effectiveness [19] but overall structure also plays a role and impacts myosin kinetics [20]. Each of these substitutions is expected to affect myosin kinetics related to its interaction with actin, e.g. actin-activated ATPase and motility. Heterozygous Q371P alleles are significantly abundant although no homozygous individuals were detected. Abundance of the other SNPs is untested.

Loop 2 and near Loop 2 SNP substitutions are A621V, T631N (E631 in MYH1), A637V (G637 in MYH1), and E643K (K643 in MYH1). A/V substitution replaces the apolar alanine with the larger apolar valine, T/N exchanges two neutral polar groups, and E/K reverses charge. The effect of Loop 2 on myosin ATPase and myosin interaction with actin has been thoroughly studied with site directed mutagenesis. In Dicty myosin II, Loop 2 tolerates significant sequence changes without affecting function. Function seems to depend on charged or apolar residue distribution but not on specific structural features because the loop is flexible. A/V and T/N substitutions are conservative and unlikely to impact function. The A637V substitution is from perinatal myosin and abundant in this isoform although no homozygous individuals were detected. Abundance of the other Loop 2 SNPs is untested.

The Myopathy Loop is a structured surface loop containing an unusual clustering of heart disease implicated mutations including R406Q/W/L, V407M, V409M, and G410V [27]. It was shown to be an actin binding site [69,92] and modulator of actin activated ATPase [81,93]. At R406, the smooth muscle heavy meromyosin (HMM) model for β-cardiac myosin has largely reiterated the clinical phenotype [94]. The G410V mutant shows modest changes in actomyosin affinity possibly indicating a generally degraded fit between myosin and actin like the R406 mutants [20]. The R406W mutant appears in the SNP data base in apparently healthy individuals. R408C (K408 in MYH1) has not been implicated in disease. None of the Myopathy loop SNPs have significant abundance although homozygous individuals carrying R406W were identified.

The Loop 3 SNP substitutions, R570K (K570 in MYH1) and V572A/D (A572 in MYH1), and the hydrophobic actin binding site residue substitutions, F534L and F655S, are conservative except for F655S. Among the myosin isoforms, all of these residues are variable. The heterozygous allele for F534L in skeletal myosin 2b has significant abundance (2.8%) but no homozygous individuals were identified.

### Other myosin S1 SNPs

SNPs appearing in Table 1 but not discussed in the context of Figures 2, 3, 4, 5, and 6 locate to the N-terminus (AA1-219), the upper 50 k (AA220-468) and lower 50 k (AA469-620). The upper 50 k and lower 50 k domains (U50 and L50) designate peptides within the 50 k of S1 on either side of the cleft that closes on strong binding to actin. N-term SNP substitutions include D4A in MYH7 and D4E in MYH13, R18C, R24P/L, T25M (I25 in MYH1), M142I (A142 in MYH1), R145G (G145 in MYH1), I148T (R148 in MYH1), and L199F (I199 in MYH1). The U50 SNP substitutions include H287R, E300D (L300 in MYH1), T307P, P320A (E320 in MYH1), S323C (V323 in MYH1), I326T, V335I (T335 in MYH1), R336H (D335 in MYH1), T345A, V350D/A from myosin 2b and R350W (V350 in MYH1) from myosin X. L50 SNP substitutions are I514T and A594T. SNP substitutions Q329R and I514T are disease implicated. SNPs in the "other myosin S1" category do not fall within, or are not spatially proximal to, presently recognized S1 functional elements directly participating in transduction. The average heterozygous SNP allele frequency for this set ranges from 0 to 17.2% (17.2% for E389D in myosin V with homozygous individuals identified) with most heterozygous substitutions having significant abundance and some untested.

### Identifying sites for probing motor dynamics

**Figure 7:**
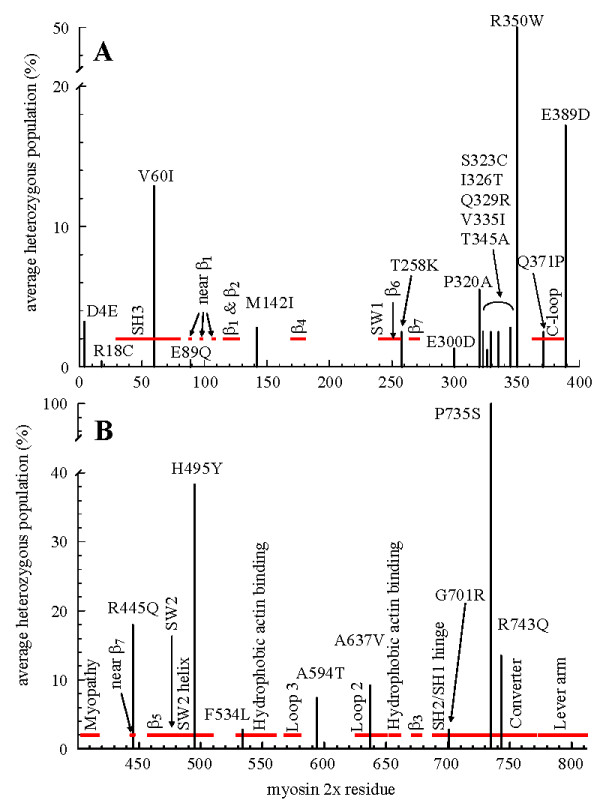
**Function-neutral candidate SNP missense substitutions in myosin heavy chain**. The x-axis is the myosin 2× (MYH1) sequence from 1-400 in **Panel A **and 401-812 in **Panel B**. The y-axis indicates AHP in percent. Substitutions with AHP ≥ 0.5% are denoted by a vertical bar and by the residue substitution and sequence number in a horizontal label. The thick broken horizontal red line indicates myosin functional domain positions described in the text. Domain names are denoted with vertical labels. Converter domain (**Panel B**) SNP substitution P735S has an AHP showing 100% substitution in the 4 subpopulations tested suggesting the reference sequence is incorrect.

Figure 7 shows the amino acid sequence of myosin 2× vs AHP for the SNPs in Table 1. Myosin regions or domains identified with specific functions in energy transduction are identified by the broken red line and with a vertical label. Many SNPs have unknown or zero AHP that are both plotted as zero. The remaining substitution sites have a significant-to-large AHP and are identified with a horizontal label. Healthy individuals with homozygous SNP substituted alleles are implied by the significant heterozygous populations. Figure 7A, specifying SNPs in the AA1-400 portion of the MHC show numerous substitutions at the N-terminus and in the AA280-360 peptide segments. These regions are not directly related to core function because they do not fall into the identified functional subdomains. The C-loop and β_6_-β_7 _junction are functional elements that are the exceptions in this region because they are SNP modified. Figure 7B, specifying SNPs in the AA401-813 portion of the MHC, shows a somewhat different picture with all but one significant SNP substitution modifying a functional sub-domain. The high AHP sites are candidates for modification by mutagenesis to introduce probes that monitor motor dynamical structure without altering native behavior. Most crucial regions are covered where in introduction of Trp or Cys would facilitate direct (Trp) or indirect, through a specific modification by an extrinsic probe (Cys), observation of myosin dynamics. Remarkably, the G701R substitution in the SH2/SH1 hinge falls into this category.

## Discussion

SNPs account for about 75% of the number of sequence differences between individuals in a population [95]. They have significant utility in gene mapping studies and there are an increasing number of SNPs that have been shown to have a significant influence on an individual's susceptibility to disease and response to various drug therapies. In some well-studied genes and gene families, patterns of SNPs within the coding sequences have also provided some insight into protein function. Perhaps the best known example of this is the SNP (rs334) that leads to sickle cell anemia. This Glu6Val change in the protein sequence has an allele frequency of about 25% in Yoruban populations and creates well-known negative and presumed positive consequences for the individuals that carry this sequence variant. Myosin SNPs could likewise create negative and positive consequences for certain populations with specific functional requirements that should be explored experimentally. Nonsynonymous SNPs that have truly neutral functional consequences also provide important information about protein function, because they highlight regions of protein structure that are tolerant of structural variation. We discuss three scenarios for interpreting SNP data denoted, the function-neutral, the too-robust, and the too-sensitive.

SNPs reside in healthy people, otherwise they are deleted by natural selection, hence it might be hypothesized that the amino acid substitution is function-neutral. In this case, SNPs identify residues or components of the motor not directly related to core function making them candidate sites for adventitious modification. An example is the R240W substitution that places a spectroscopic probe in the 7-stranded β-sheet at the interface of the active and actin-binding sites. It is thought that coordination of ATP hydrolysis with actin binding and release is mediated by this β-sheet and an optical probe of its structure would be valuable. R240 is not highly conserved among species but infrequently substituted by aromatic residues. Its negligible AHP raises uncertainty about potential use although homozygous individuals were reported. G701 is the pivot for the lever-arm swing and thought to be necessary for myosin function. The G701R SNP substitution has 2.8% AHP but it is difficult to understand how this substitution could be function-neutral in the swinging lever-arm picture for force production. Other unlikely function-neutral candidates are the R246H and E469Q substitutions with unknown and insignificant AHP, respectively. R246 and E469 form the SW1/SW2 salt-bridge regulating phosphate release in ATPase in the rate limiting step for muscle myosins. R246H originates from the cellular myosin VII isoform where the native rate limiting ADP release minimizes R246 significance. E469Q originates from β-cardiac myosin using the phosphate release mechanism. Under the function-neutral hypothesis, the collection of SNPs at R246, E469 and the vicinity of the 7-stranded β-sheet would imply a diminished role for this structure in energy transduction.

Figure 7 identifies the most likely candidates for the function-neutral SNPs because they have significant to large AHP implying the presence of healthy individuals carrying homozygous substituted alleles. Here SNPs cluster in a few regions of the myosin head not identified with core functionality but also cover most function-crucial regions in the S1. SNPs in Figure 7 may be most useful as leads for the introduction of probes that monitor motor dynamical structure without altering native behavior.

An alternative to the function-neutral hypothesis is that SNP substitutions modify functional structures too-robust to be disturbed by otherwise intrusive sequence changes. Excluding for a moment the E469Q and G701R special cases, several structures in S1 fulfill expectations implied by this hypothesis. The 7-stranded β-sheet and vicinity collected 17 SNP substitution sites (excluding known disease related sites, see Table 1) over 14 of the 22 myosin genes where SNPs were identified. The too-robust hypothesis implies that the energy transduction mechanism contained in the 7-stranded β-sheet is too well designed by evolution to be compromised by any of the SNP substitutions. Actin binding by S1, modified by SNPs in the main hydrophobic binding site (F534L and F655S), in Loop 3 (R570K and V572A/D), Loop 2 (A621V, T631N, A637V, and E643K), the Myopathy loop (R408C, not including the disease implicated R406W), and the C-loop (R370H, Q371P and P378Q), is another potential too-robust site. The switch 2 helix, charged with propagating linear motion from the active site to the converter domain, suffers seven SNP substitutions and functions while the SH2/SH1 hinge and lever-arm successfully manage impacts of six and two SNP substitutions, respectively. Returning to E469Q and G701R, the functional impact of E469Q (7-stranded β-sheet) has not been evaluated in vitro, however, other substitutions at this site were disruptive to muscle myosin function except when the polarity of the R246/E469 salt-bridge was reversed. Then the mutant S1 functioned near normally. The E469Q substitution seems likely to disrupt the interaction with R246 thereby altering specific rates in the ATPase cycle but potentially within boundaries that do not overly impact function. The G701R substitution is difficult to rationalize even in the too-robust hypothesis. The functional impact of G701R has likewise not been evaluated in vitro but a glycine swivel is disrupted by the presence of any side chain with a β-carbon. How super-fast myosin could function with G701R needs clarification.

The too-robust hypothesis is often the implicit working hypothesis for those utilizing site directed mutagenesis to study protein mechanisms. It may sometimes be too optimistic an assumption for interpreting SNPs. The SNP substitution may affect protein function compensated by redundant functionality outside the affected protein in the larger physiological system. Different SNPs will have different applicable hypotheses and the challenge is to identify the correct one to apply.

**Figure 8:**
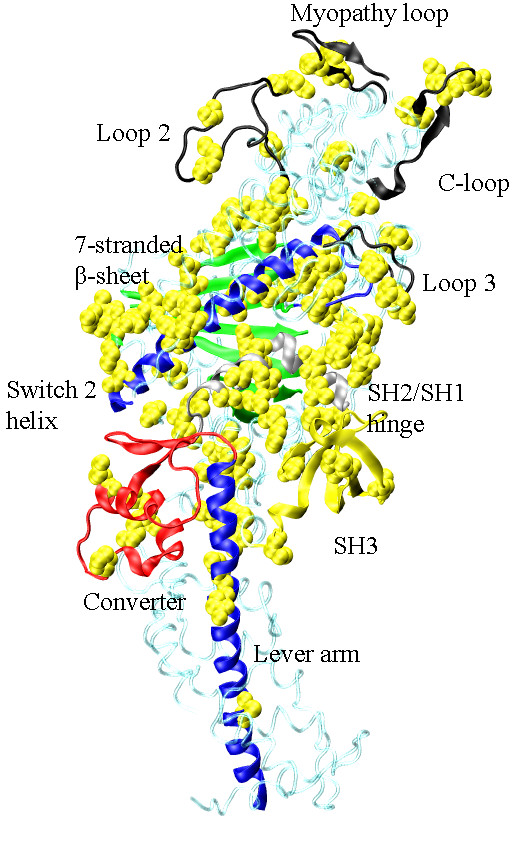
**The S1 crystal structure depicted in Figure 1 and with the heavy chain SNP substitution sites indicated with space filling models of the unsubstituted side chains**. SNP substitutions locate throughout the myosin S1 heavy chain.

We anticipated SNP clustering in a few regions of the myosin head identifying the expendable components of the system unrelated to core functionality. This occurred to some extent within the narrow context of the significant AHP SNPs. Residues 1-400 in MHC show significant AHP SNP substitutions at the N-terminus and in the AA280-360 peptide segments that do not fall into the identified functional sub-domains. However, residues 401-812 show a different picture with all but one significant AHP SNP substitution modifying functional sub-domains. Overviewing the total set of substitutions, the S1 SNPs are distributed spatially over the protein more uniformly than expected. Figure 8 shows the annotated S1 from Figure 1 with the SNP substitution sites identified by the solid atom rendering in yellow. Most SNPs locate within, or to the vicinity of, domains intimately connected to the myosin core functionality. They do not seem to select any one structural feature for a diminished role in the core functionality except possibly the 7-stranded β-sheet that attracted so many SNP substitutions.

The Myopathy loop and the converter domain nearly escaped modification by SNPs not already disease related. They each have one SNP substitution not known to be disease implicated. As suggested by its name, known mutations in the Myopathy loop accompany heart disease [27]. SNPs do not appear in the Myopathy loop because most substitutions cause serious functional deficit. The same appears to be true for the converter where there are 10 or more heart disease linked mutations. These structures are too-sensitive to substitution to be a target for SNPs. The large AHP for the SNP substitution R743Q in the converter domain (Figure 7) appears to have identified a unique site in the domain that can be safely modified. At least for myosin, regions or domains excluded from SNP substitution may be the most precisely defined. These high sensitivity regions are anti-targets for SNPs because they are not functionally robust (i.e. too-sensitive) rather than because they are the only domains necessary for function.

## Conclusions

Single nucleotide polymorphisms of the myosin heavy chain were mined from the NCBI data base using computer programs written in perl that identified human nonsynonymous SNP amino acid missense substitutions for any MHC gene. Twenty-two MHC genes were searched including muscle and non-muscle myosin isoforms. Excluding disease implicated residues and sites without homology with skeletal myosin 2×, the 71 SNPs identified were distributed spatially homogeneously over MHC with 22 falling outside specific functional domains. The remaining 49 SNPs were spatially related to the 7-stranded β-sheet mediating active site and actin binding site communication (17 SNPs), the actin binding peptides (12 SNPs), switch 2 helix (7 SNPs), SH2/SH1 hinge (6 SNPs), SH3 (4 SNPs), lever-arm (2 SNPs), and converter (1 SNP). Two functional elements were almost devoid of SNPs, the converter domain and the Myopathy loop. These elements are burdened with an unusually high number of disease implicated mutations. The SNP substitution sites in MHC suggest they can infiltrate domains that are engineered by evolution to be "too-robust" to be disturbed by otherwise intrusive sequence changes, and, "function-neutral" sites where substitution is immaterial. Disease implicated mutations and the absence of SNP substitutions map to regions "too-sensitive" to be modified because they have not evolved a robust sequence paradigm for performing their function.

## Methods

### Automated SNP retrieval

SNP data records were retrieved using a computer program written in perl. Perl was chosen because of its text-parsing and internet capabilities; and because it is widely available. The basic distribution of perl is available for use with various operating systems, including Microsoft Windows, linux/unix, and Macintosh. The first program (get_snps_flt.pl) uses NCBI eUtils to search for missense SNPs in the human myosin genes. The results are saved locally as flat text files then parsed into tables using a second perl program (make_table.pl). The get_snps_flt.pl and make_table.pl programs are listed in the additional file 1 where there are instructions for their use.

The NCBI SNP database stores individual SNP submissions and assigns them unique identifiers that are prefixed with ss (submitter sequence). These individual records are eventually assigned to groups so that all SNPs that occur at a particular position in a gene are in the same cluster. Each cluster has a unique numeric identifier that is prefixed with rs (reference sequence). Table 2 shows a representative tabular output from make_table.pl. In every cluster, the reference (or "normal") allele is compared to the SNP substituted allele to determine the missense mutation in the expressible protein. Up to three SNPs may be reported in a cluster, corresponding to the three possible non-reference bases. The residue sequence in Table 2 is the sequence of the gene product, or myosin isoform, in which the SNP is detected. Sequence alignment with BLAST positioned the SNP substitutions on the myosin crystal structure.

**Table 2:** SNPs for smooth muscle myosin MYH11

**ID of SNP Cluster**	**Gene**	**Function**	**Base Change**	**Amino Acid Change**	**Peptide Sequence#^a^**
rs61734198^b^	MYH11	missense	G -->C	Q -->H	473
	MYH11	missense	G -->T	Q -->H	473
	MYH11	missense	G -->C	Q -->H	480
	MYH11	missense	G -->T	Q -->H	480
rs16967494	MYH11	missense	G -->A	A -->T	1234
	MYH11	missense	G -->A	A -->T	1241
rs16967494	MYH11	missense	G -->A	A -->T	1234
	MYH11	missense	G -->A	A -->T	1241
rs35176378	MYH11	missense	A -->G	M -->V	1508
	MYH11	missense	A -->G	M -->V	1515
rs35035518	MYH11	missense	C -->T	S -->L	883
	MYH11	missense	C -->T	S -->L	890
rs34321232	MYH11	missense	A -->C	K -->Q	1621
	MYH11	missense	A -->C	K -->Q	1628
rs34263860	MYH11	missense	G -->A	A -->T	1104
	MYH11	missense	G -->A	A -->T	1111
rs16967510	MYH11	missense	T -->C	V -->A	1289
	MYH11	missense	T -->C	V -->A	1296
rs16967494	MYH11	missense	G -->A	A -->T	1234
	MYH11	missense	G -->A	A -->T	1241
rs12149651	MYH11	missense	C -->A	L -->M	1053
	MYH11	missense	C -->A	L -->M	1060
rs7196804	MYH11	missense	G -->A	V -->M	1310
	MYH11	missense	G -->A	V -->M	1317
rs1801902	MYH11	missense	A -->G	T -->A	864
	MYH11	missense	A -->G	T -->A	871

^a^SNPs corresponding to amino acid residues in the tail portion of myosin appear in the output for the automated SNP retrieval program but lie outside the motor domain of myosin and are not discussed in the text.

^b^SNP rs61734198 is an example of a two base change (G→C and G→T) SNP where His substitutes for Gln in both cases. The MYH11 gene product includes splicing variants with different sequences hence the same SNP has two sequence numbers in the variants that are in this case sm1A (AA473) and sm2B (AA480).

### Monte Carlo Molecular Dynamics (MCMD) simulation of myosin dynamics

The dominant kinetic pathway for the actomyosin ATPase cycle is summarized by solid arrows in Scheme 1.

M, M*, M**, and M^ are intermediates corresponding to distinct myosin conformations, A is actin, T is ATP, D is ADP, and P is inorganic phosphate. M* and M** weakly bind actin while M and M^ strongly bind actin. The broken arrow (···>) in the bottom row indicates the rate limiting product release step in the absence of actin. Work production occurs when M** ·D·P weakly binds actin (vertical transition), releases product P with the opening of the active site "back door" (R246/E469 salt-bridge), and forms the strong actin bond A·M^·D. The lever-arm rotates to impel actin ending in the low free energy rigor state A.M. Cross-bridge repriming begins with ATP binding to A.M initiating dissociation from actin, ATP hydrolysis, and reversal of the lever-arm power stroke rotation. The power stroke, envisioned by comparison of the M** and M* crystal structures, rotates the lever-arm through ~70 degrees [17,53]. We performed a dynamics simulation for myosin joining the known M** and M* transient intermediate structures in the Scheme 1pathway using a non-equilibrium Monte Carlo molecular dynamics (MCMD) simulation of the M** → M* conformation trajectory [19].

The lever-arm swing conformation change in myosin occurs on the millisecond time domain and involves many atoms. We developed the new dynamics simulation strategy because system complexity excludes use of standard molecular dynamics methods. All conformation trajectories confirm that during ATPase, an entropy dominated structural transition in the actin binding domain of S1 is a free energy barrier ensuring that product release is rate limiting in the absence of actin and prior to the lever-arm swing in agreement with the conventional myosin mechanism. MCMD simulation of the power stroke indicated that two complementary peptides, designated U50a and U50b (AA145-361 and AA362-462 peptides), mainly in the 50 kDa actin binding domain encompass the free energy barrier to product release and the anti-barrier, respectively. Simulation shows that coupling the U50a and U50b conformation transitions removes the free energy barrier to product release. The C-loop is a structured surface loop linking U50a and U50b whose structure would be perturbed with actin binding. These circumstances suggest that perturbation of the C-loop with actin binding couples U50a and U50b transitions causing product release and power stroke initiation.

### Homology Modeling

Homology modeling uses a crystal structure template to constrain a target sequence of unknown structure. The template is the chicken skeletal myosin S1 (2mys, [17]) and the target sequence is the human fast skeletal MHC isoform 2× (MYH1) and essential light chain 3 (ELC3). Homology modeling was done with Modeller 9.4 [96]. All structures shown depict S1 from myosin 2×.

Accurate model building depends on target and template sequence similarity and alignment. Template and target sequences for the MHC and ELC are practically identical (>91% sequence identity) suggesting Modeller produces a reliable structure [96]. We did alignment using the NCBI BLAST 2 sequences protocol http://www.ncbi.nlm.nih.gov/blast/bl2seq/wblast2.cgi. Unstructured surface loops on myosin were not resolved in the crystal structures but were added using the appropriate Modeller routine. Homology model accuracy was evaluated using the Discrete Optimized Protein Energy (DOPE) score as suggested in the Modeller tutorial. The DOPE score is proportional to potential energy per residue, smoothed over a 15 residue window, and normalized by the number of restraints acting on each residue. It indicates problem regions in the homology model when profiles are compared from target and template sequences. The DOPE profile showed no unusual problem regions over the entire peptide.

Visualizations of myosin structures were created in Visual Molecular Dynamics (VMD) [97] then rendered in POV-Ray http://www.povray.org/ and output as bitmap files.

## Abbreviations

(AA): amino acid; (AHP): average heterozygous population; (ASA): accessible surface area; (βS1): β-ventricular myosin subfragment 1; (Dicty): *Dictyostelium discoideum*; (DOPE): discrete optimized protein energy; (ELC): myosin essential light chain; (EOM): extraocular muscle; (L50): lower 50 k domain of S1 containing AA469-620; (LC1): long ELC isoform; (LC3)short ELC isoform; (MHC): myosin heavy chain; (MLC): myosin light chain; (NCBI): National Center for Biotechnology Information; (RLC): myosin regulatory light chain; (S1): myosin subfragment 1; (SAH): single α-helix; (SNP): single nucleotide polymorphism; (U50): upper 50 k domain of S1 containing AA220-468; (U50a): U50 sub-domain (AA145-361): containing free energy barrier to phosphate release in myosin ATPase; (U50b): U50 sub-domain (AA362-462): containing free energy anti-barrier to phosphate release in myosin ATPase

## Authors' contributions

TPB performed the SNP searches, aligned protein sequences, and drafted the manuscript. KLN wrote the perl software and adapted it for use on the project. EDW and KA provided conceptual guidance and helped draft the manuscript. All authors read and approved the final manuscript.

## Supplementary Material

Additional file 1 **Instructions for retrieving SNPs**. Two perl programs provide automated retrieval and organization of SNP data from the NCBI database. Additional file 1 contains the perl programs and instructions for their use. Click here for file
