# Supplementary material for: Myosin individualized: single nucleotide polymorphisms in energy transduction
Source: BMC Genomics. 2010 Mar 15;11:172. doi: 10.1186/1471-2164-11-172 (PMC2848645; doi:10.1186/1471-2164-11-172)
Supplement: Additional file 1 — Instructions for retrieving SNPs. Two perl programs provide automated retrieval and organization of SNP data from the NCBI database. Additional file 1 contains the perl programs and instructions for their use. [file 1471-2164-11-172-S1.DOC]

SUPPORTING INFORMATION

*Instructions for retrieving SNPs*.

SNPs are simple to retrieve from the NCBI data base using the NCBI Web interface. In the Search drop down box select SNP and in the “for” window type “MYH11 human missense” for the smooth muscle myosin heavy chain missense mutation SNPs. The Web browser will return a listing of the SNPs on record. The list provides the rs numbers and some other graphical information. If you click on the rs number you will see all of the information and links related to the SNP.

A better automated search method uses the two perl programs, get_snps_flt.pl and make_table.pl, listed below. On a PC, download the free perl software (<http://www.perl.com/download.csp>) and install it on your machine.

1. Make a new directory to contain your perl programs and their output then cut the perl programs one at a time from the listings below and paste them into new files named get_snps_flt.pl and make_table.pl.

2. Open a DOS window for running the perl programs by using Run from the Start menu then type cmd.

3. In the DOS window, move to the directory where you have the perl programs and where your output should go then type:

perl get_snps_flt.pl

This will start the SNP retrieval program for the genes listed in the perl code (see below). Change these entries to suit your interests. The program will create a new file for each gene using the gene name. They will have names like “snp_MYH11_human_missense.dat”.

4. When get_snps_flt.pl finishes, type:

perl make_table.pl snp_MYH11_human_missense.dat>fMYH11.dat

This will create a file named fMYH11.dat containing data formatted like that in **Table 1** from the information in snp_MYH11_human_missense.dat.

Listing for get_snps_flt.pl

#!/usr/bin/perl -w

use strict;

use LWP::UserAgent;

use HTTP::Headers;

## Get all SNP records from NCBI for your gene.

##

##

my $base =

"http://eutils.ncbi.nlm.nih.gov/entrez/eutils/esearch.fcgi?" .

"db=snp".

"&tool=get_snps_flt.pl" .

"&email=neff.kevin%40mayo.edu";

# Autoflush the output buffer

$| = 1;

# Create a "browser"

my $hdr = new HTTP::Headers( 'Accept' => 'http', 'User-Agent' => 'Mozilla/1.0' );

my $ua = new LWP::UserAgent;

# Replace these strings with the searches you want to run. Specify the

# search just like you would if you were using Entrez

my @genes = ( "MYO1E human missense",

"MYO6 human missense",

"MYO7A human missense",

"MYO7B human missense",

"MYO9A human missense",

"MYO9B human missense",

"MYO10 human missense",

"MYH1 human missense",

"MYH2 human missense",

"MYH3 human missense",

"MYH4 human missense",

"MYH6 human missense",

"MYH7 human missense",

"MYH8 human missense",

"MYH9 human missense",

"MYH10 human missense",

"MYH11 human missense",

"MYH12 human missense",

"MYH13 human missense",

"MYH14 human missense",

"MYH15 human missense" );

# Iterate over list of genes. For each, collect all IDs for matching records.

for( my $m = 0; $m <= $#genes; $m += 1 ) {

# File where the results will be stored (per query)

my $filename = $genes[$m];

$filename =~ s/ /_/g;

# This first search is used mainly to get the number of search results.

printf( "# Searching for `$genes[$m]`\n" );

my $retmax = "&retmax=";

my $url = $base . "&term=$genes[$m]";

$url =~ s/\[/%5B/g;

$url =~ s/\]/%5D/g;

$url =~ s/"/%22/g;

$url =~ s/ +/\+/g;

my $req = new HTTP::Request( "GET", $url, $hdr );

my $got = $ua->request( $req );

if( ! $got->is_success ) {

print( STDOUT "Error: $url\n" );

exit( 10 );

}

else {

# Figure out how many records to request to get all IDs in one chunk

my @lines = split( /\n/, $got->content );

for( my $j = 0; $j <= $#lines; $j += 1 ) {

if( $lines[$j] =~ /Count/ ) {

$lines[$j] =~ s/ +//;

$lines[$j] =~ s/^.*<Count>//;

$lines[$j] =~ s/<\/Count>.*$//;

$retmax .= $lines[$j];

last;

}

}

}

# Download all of the IDs at once and put them in an array.

my $id_index = 0;

my @ids = ();

$url .= $retmax;

$req = new HTTP::Request( "GET", $url, $hdr );

$got = $ua->request( $req );

if( ! $got->is_success ) {

print( STDOUT "Error: $url\n" );

exit( 10 );

}

else {

my @lines = split( /\n/, $got->content );

for( my $j = 0; $j <= $#lines; $j += 1 ) {

if( $lines[$j] =~ /Id>/ ) {

$lines[$j] =~ s/ +//;

$lines[$j] =~ s/^.*<Id>//;

$lines[$j] =~ s/<\/Id>.*$//;

$ids[$id_index] = $lines[$j];

$id_index += 1;

}

}

}

printf( STDERR "IDs downloaded: %d\n", ($#ids+1) );

if( $#ids == -1 ) {

printf( STDERR "A Terrible Error has occured. Skipping.\n" );

next;

}

# Open the file to which summaries will be dumped

open( MORE, ">snp_$filename.dat" )

or die( "Cannot open file ($filename) for output" );

# Base address for getting more information about a particular SNP.

my $base_moreinfo = "http://eutils.ncbi.nlm.nih.gov/entrez/eutils/efetch.fcgi?" .

"db=snp" .

"&report=FLT" .

"&tool=get_snps_flt.pl" .

"&email=neff.kevin%40mayo.edu";

# start downloading summaries, in batches, pausing now and then

# as a throttle.

my $summary_line = "";

for( my $i = 0; $i <= $#ids; $i += 1) {

sleep rand()*2;

my $ids_request = "&id=$ids[$i]";

print( STDERR "$ids[$i]\n" );

do {

$url = "${base_moreinfo}&id=$ids[$i]";

$req = new HTTP::Request( "GET", $url, $hdr );

$got = $ua->request( $req );

if( ! $got->is_success ) {

print( STDERR "\nFailed!\n" );

print( STDERR "Error: $url\n" );

sleep( 30 );

}

} while( ! $got->is_success );

my @lines = split( /\n/, $got->content );

foreach my $line (@lines) {

print( MORE "$line\n" );

}

}

}

close( MORE );

Listing for make_table.pl

#!/usr/bin/perl -w

use strict;

## This program extracts information from data downloaded from the NCBI

## database of SNPs, formatted as flat files (FLT). Use it in conjunction

## with get_snps_flt.pl.

##

# Removes labels from

sub remove_labels($)

{

my $str = $_[0];

$str =~ s/\|//g;

$str =~ s/ [^= ]*=/ /g;

$str =~ s/[ \t]+/ /g;

return $str;

}

# Table Headings

printf( " ID of SNP Gene Function Base Residue Residue \n" );

printf( " Cluster Change Change Sequence # \n" );

printf( "---------------------------------------------------------------------\n" );

my $assembly_count;

my $rs = "";

while( <> ) {

chomp();

# Get cluster ID

if( /^rs[0-9]/ ) {

s/ .+$//;

$rs = $_;

$assembly_count = 0;

}

# Count the number of assemblies in this cluster

while ( /CTG/ && /assembly/ ) {

$assembly_count += 1;

$_ = <>;

chomp();

}

# Get all lines from this record that record a SNP

my @lines = ();

while ( /LOC/ && /aa_position/ ) {

push( @lines, remove_labels($_) );

$_ = <>;

chomp();

}

# Find the first reference record for this cluster. There may be

# several reference records (which may differ in position, depending

# on which RNA model is used) but I only need to know the gene, the

# base that's normally found in this position of the genome, and the

# reference amino acid. These values do not seem to differ from one

# reference line to the next.

my @ref;

foreach my $l (@lines) {

if( $l =~ /reference/ ) {

@ref = split( /[ \t]+/, remove_labels($l) );

last;

}

}

# Print a line in the table for each SNP at this location

my $count = 0;

my @lines_to_print;

foreach my $l (@lines) {

if( $l !~ /reference/ ) {

my @snp = split( /[ \t]+/, remove_labels($l) );

my $str = sprintf( " %5s %10s %1s --> %1s %1s --> %1s %4i\n",

$ref[1], $snp[3], $ref[4],$snp[4], $ref[6],$snp[6], $snp[7] );

push( @lines_to_print, $str );

$count += 1;

}

}

# Remove what appear to be repeats. These records appear to be

# repeated because the FLT-formatted output of the NCBI SNP

# database is a subset of the record. These identical records

# generally reflect identical (in allele and position) using

# several RNA models.

my %freq;

foreach my $l (@lines_to_print) {

$freq{ $l } += 1;

}

my @uniq = keys( %freq );

# Sort each cluster by position. This makes it easier to see

# which alleles also have different positions in the protien

my @uniq_sorted = sort {

my @alist = split(' ', $a);

my @blist = split(' ', $b);

$alist[-1] <=> $blist[-1];

} @uniq;

# Print lines in table, one line per allele (or position)

for( my $i = 0; $i <= $#uniq_sorted; $i += 1 ) {

if( $i == 0 ) {

printf( "%12s", $rs );

}

else {

printf( "%12s", "" );

}

print( $uniq_sorted[$i] );

}

}
